# Identified γ-glutamyl cyclotransferase (GGCT) as a novel regulator in the progression and immunotherapy of pancreatic ductal adenocarcinoma through multi-omics analysis and experiments

**DOI:** 10.1007/s00432-024-05789-0

**Published:** 2024-06-25

**Authors:** Ying Zheng, Qunli Xiong, Yang Yang, Yifei Ma, Qing Zhu

**Affiliations:** Division of Abdominal Tumor Multimodality Treatment, Cancer Center, West China Hospital, Sichuan University, No.37 Guoxue Alley, Chengdu, 610041 Sichuan China

**Keywords:** Pancreatic ductal adenocarcinoma (PDAC), GGCT, Immunotherapy, Pan-cancer

## Abstract

**Background:**

Pancreatic ductal adenocarcinoma (PDAC) is renowned for its formidable and lethal nature, earning it a notorious reputation among malignant tumors. Due to its challenging early diagnosis, high malignancy, and resistance to chemotherapy drugs, the treatment of pancreatic cancer has long been exceedingly difficult in the realm of oncology. γ-Glutamyl cyclotransferase (GGCT), a vital enzyme in glutathione metabolism, has been implicated in the proliferation and progression of several tumor types, while the biological function of GGCT in pancreatic ductal adenocarcinoma remains unknown.

**Methods:**

The expression profile of GGCT was validated through western blotting, immunohistochemistry, and RT-qPCR in both pancreatic cancer tissue samples and cell lines. Functional enrichment analyses including GSVA, ssGSEA, GO, and KEGG were conducted to explore the biological role of GGCT. Additionally, CCK8, Edu, colony formation, migration, and invasion assays were employed to evaluate the impact of GGCT on the proliferation and migration abilities of pancreatic cancer cells. Furthermore, the LASSO machine learning algorithm was utilized to develop a prognostic model associated with GGCT.

**Results:**

Our study revealed heightened expression of GGCT in pancreatic cancer tissues and cells, suggesting an association with poorer patient prognosis. Additionally, we explored the immunomodulatory effects of GGCT in both pan-cancer and pancreatic cancer contexts, found that GGCT may be associated with immunosuppressive regulation in various types of tumors. Specifically, in patients with high expression of GGCT in pancreatic cancer, there is a reduction in the infiltration of various immune cells, leading to poorer responsiveness to immunotherapy and worse survival rates. In vivo and in vitro assays indicate that downregulation of GGCT markedly suppresses the proliferation and metastasis of pancreatic cancer cells. Moreover, this inhibitory effect appears to be linked to the regulation of GGCT on c-Myc. A prognostic model was constructed based on genes derived from GGCT, demonstrating robust predictive ability for favorable survival prognosis and response to immunotherapy.

**Supplementary Information:**

The online version contains supplementary material available at 10.1007/s00432-024-05789-0.

## Introduction

According to the latest data released by American National Cancer Institute, pancreatic cancer (PC) currently become the third leading cause of cancer-related deaths in the United States (National Cancer Institute 2020). Nearly 90% of pancreatic cancers manifest as pancreatic ductal adenocarcinomas (PDACs). Global patterns indicate an increasing trend in PDAC, it is anticipated to become the second most common cause of cancer-related mortality in Western nations in a near future (Siegel et al. 2023). In China, the incidence of pancreatic cancer is on the rise due to an aging population, shifts in dietary habits, and escalating life stresses. The overall 5-year survival rate for patients barely exceeds 10% (Ju et al. 2023; He et al. 2024). The majority of PDAC patients are diagnosed with distant metastasis, making it improbable that the outcome would be affected by the removal of the primary lesion through a major surgical procedure. Therefore, it is imperative to seek new therapeutic targets for pancreatic cancer.

Cancer cells often produce significant amounts of reactive oxygen species (ROS) to facilitate their rapid advancement, However, elevated levels of ROS can lead to DNA damage, resulting in cell death (Srinivas et al. 2019). In order to sustain rapid proliferation, the antioxidative system of tumor cells is enhanced to alleviate the damage induced by ROS (Hatem et al. 2017). Glutathione (GSH) is a vital component of the cellular antioxidative system, its relative high concentration in cancer cells often considered a potential biomarker for the diagnosis and treatment of tumors (Niu et al. 2021). The hypothetical protein C7orf24, identified as γ-glutamyl cyclotransferase (GGCT), facilitates the conversion of γ-glutamyl dipeptides to 5-oxoproline (pyroglutamic acid), and may potentially playing a pivotal role in regulating glutathione homeostasis (Oakley et al. 2008). Extensive research indicates that GGCT is highly expressed in various tumors and correlates with patient prognosis, high level of GGCT expression also shares a close relationship with the proliferation of cancer cells (Zhang et al. 2006; Azumi et al. 2009; Gromov et al. 2010; Takemura et al. 2014). Zhang et al. (2022) found that the interaction between GGCT and MRPL9 was involved in progression of thyroid cancer, and knockdown of GGCT and MRPL9 suppressed the growth and lung metastases of thyroid cancer in vitro and in vivo by inhibiting the MAPK/ERK signaling pathway. Another research has shown that GGCT was identified as a target of oncogenic Ras which is in need for oncogenic Ras-induced primary mouse cell proliferation and transformation and in vivo lung cancer formation in the LSL-Kras G12D mouse model (He et al. 2019). Despite GGCT being confirmed to be associated with tumorigenesis, its expression profile and biological functions in pancreatic cancer remain unexplored.

Building upon the significant biological role of GGCT in pan-cancer, our research aims to further investigate its critical involvement in the diagnosis and management of pancreatic cancer. In our study, we confirmed the high expression of GGCT in PDAC at both mRNA and protein levels, and analysis of patient survival data revealed that those with elevated GGCT expression experienced poorer prognosis. Furthermore, we employed various bioinformatics analysis methods to explore the biological functions of GGCT in PDAC, as well as its significant regulatory effects on the immune microenvironment of PDAC. We also constructed a prognostic model using GGCT-related genes, which exhibited strong predictive ability for patient overall survival (OS) and response to immunotherapy. Additionally, we observed that suppressing GGCT markedly hindered the proliferation and invasion capabilities of pancreatic cancer cells both in vitro and in vivo.

Moreover, this inhibitory effect may be achieved via the modulation of c-Myc.

## Materials and methods

### PDAC clinical samples

A total of 57 PDAC specimens were collected from patients clinically and pathologically diagnosed with pancreatic ductal adenocarcinoma. These patients had undergone surgical excision at West China Hospital, Sichuan University (Chengdu, China) between 2014 and 2018. Clinical characteristics of the above PDAC patients including age, gender, TNM stage (Following the staging criteria outlined in the 2018 eighth edition of the National Comprehensive Cancer Network), tumor grade, and preoperative CA199 and CEA values, were systematically collected. No preoperative radiotherapy or chemotherapy had been administered to any of these patients. Additionally, we collected 6 pancreatic cancer tissues and paired normal tissues from West China Hospital, Sichuan University. Approval for this study was obtained from the Institutional Ethics Committee of West China Hospital, Sichuan University. Informed consent, duly authorized, was obtained from all participating patients.

### Immunohistochemistry

Immunohistochemistry (IHC) assay was carried out according to a protocol described previously (Weng et al. 2022). Briefly, the slides were incubated with GGCT antibody (Proteintech) at a dilution of 1:100 overnight, and then a goat anti-rabbit antibody (Jackson) at a dilution of 1:250 was added to slides for 40 min at room temperature. Signalstain DAB substrate kit (Cell Signaling Technology) was used to color developing. The intensity of staining was classified into four-point levels: 0, no staining; 1, weak; 2, moderate; 3, strong. The proportion of staining was scored as follows: 0, 0–5%; 1, 6–25%; 2, 26–50%; 3,  > 50%. The intensity multiplied by the proportion of each slide was computed. A product > 3 indicated high GGCT expression, while a product ≤ 3 indicated low GGCT expression.

### Cell culture

hTERT-HPNE, AsPc-1, BxPC-3, CFPAC-1, MIA PaCa-2, PANC-1 cell lines were obtained from the cell bank of the Shanghai Institute of Cells, Chinese Academy of Science (Shanghai, China). All cells were cultured respectively in DMEM, IMDM or RPMI-1640 Medium supplemented with 10% fetal bovine serum, 100 U/mL penicillin G, and 100 mg/mL streptomycin in 5% CO_2_ atmosphere at 37 °C.

### qRT-PCR

Extraction of RNA from cell lines and frozen tissues was conducted using the Trizol reagent (Invitrogen, Thermofisher, Waltham, MA, USA), following the manufacturer's instructions. Subsequent reverse transcription utilized the PrimeScript™ RT Master Mix (TaKaRa, Shiga, Japan). Real-time quantitative PCR was conducted using NovoStart® SYBR qPCR SuperMix Plus (Novoprotein, Suzhou, China). The amplification protocol included an initial denaturation at 95 °C for 30 s, followed by 40 cycles at 95 °C for 5 s, 60 °C for 30 s, and 72 °C for 30 s. Target gene expression was normalized against β-actin using the relative quantification (2^−∆∆Ct^) method. The primers used in this study were listed as follows:

β-actin:

Forward: 5′-CATGTAC GTTGCTATCCAGGC-3′;

Reverse: 5′-CTCCTTAAT GTCACGCACGAT-3′

GGCT:

Forward: 5′-AGCAACCTGCTGACAGAGAGGA-3′-;

Reverse: 5′-GGCTATCCCTCCATGCCAAGTT-3′

c-Myc:

Forward: 5′-CCTGGTGCTCCATGAGGAGAC-3′;

Reverse: 5′-CAGACTCTGACCTTTTGCCAGG-3.

18 s:

Forward: 5′-ACCCGTTGAACCCCATTCGTGA-3′;

Reverse: 5′-GCCTCACTAAACCATCCAATCGG-3′

### Western blot

Cells were seeded in six-well plates at 2.5 × 10^5^ and lysed in RIPA buffer supplemented with 1% protease inhibitor cocktail (Bimake, Houston, TX, USA). Proteins were separated by SDS–PAGE and then transferred to PVDF membranes. After blocking in 5% skimmed milk for 90 min, the membranes were incubated with primary antibodies including GGCT (Proteintech, Wuhan, China), c-Myc (Proteintech, Wuhan, China), E-cadherin (ZENBIO, Chengdu, China) and N-cadherin (ZENBIO), at 4 °C overnight. The blots were washed before the application of secondary antibodies at room temperature for 2 h. Immunocomplexes were visualized using an electrochemiluminescence reagent (Millipore, Burlington, MA, USA), with GAPDH serving as the loading control.

### Lentiviral transduction

For developing the stable GGCT knockdown and c-Myc overexpression PANC-1 and Mia Paca-2 cells, short hairpin RNAs (shRNAs) targeting the GGCT gene were designed and synthesized. The shRNA sequences were listed in the supplementary material. The plasmid overexpressing c-Myc was kindly provided by Dr. Chen Qin at Sichuan University. The Lentiviruses were generated by co-transfecting HEK293T cells with lentiviral packaging plasmids, psPAX2 and pMD2.G, together with shGGCT, OE-c-Myc, or the corresponding control shRNA pLKO.1 plasmid. The supernatant from each HEK293T culture was collected between 48 and 72 h post-transfection and subsequently filtered using a 0.45 μm filter. Following that, cells were transduced with lentiviral-containing supernatant and subjected to selection using DMEM supplemented with puromycin (2 μg/mL; Sigma-Aldrich, USA) for a duration of 48 h.

### CCK8, Edu and clone formation assay

For CCK8 assay, PDAC cells (5 × 10^3^) were cultured in 96-well plates for 0, 2, 4 days, and 10% of CCK8 reagent (TargetMol) per well were added and incubated at 37 °C for 2 h. Absorbance was measured at 450 nm using a multifunctional enzyme marker. For clone formation assay, cells (2 × 10^3^) were seeded in a 12-well plate for 7–14 days and fixed by 4% formaldehyde in PBS for 15 min, then stained with crystal violet for 25 min. Edu assays was conducted according to the protocol of Cell-Light EdU Apollo567 In Vitro Kit.

### Migration and invasion assays

PDAC cells (5–8 × 10^4^) after intervention were seeded in a transwell cell culture chambers with an 8-μm pore size polycarbonate membrane. With specificity, cells were seeded into the upper chambers with 200 μL of serum-free medium, while the lower chamber contains 800 μL complete medium containing 10% FBS. For invasion assay, Matrigel™ (Corning, Corning, NY, USA) diluted at 1:15 ratio with medium was coated in the upper chamber. After incubating in the culture chamber at 37 °C for 24 h, the upper chamber was fixed in 4% paraformaldehyde for 15 min and stained with crystal violet for 20 min.

### Xenograft model and lung metastasis model

Xenograft and lung metastasis models were established using female NCG mice (aged 6 weeks, females, five mice per group), procured from Beijing HFK Bioscience. For xenograft model, PANC-1 cells after lentiviral transduction (1 × 10^7^) along with 50 μL Matrigel™ (Corning, NY, USA) were injected into the right flanks of the randomly assigned mice, then the tumor has been growing for approximately 20 days. For in vivo lung metastasis model, PANC-1-luc-puro cells (1.5 × 10^6^) were injected via the tail vein into the mice, and imaging was conducted 20 days after the injection. All animal experiments received approval from the Institutional Animal Care and Treatment Committee of Sichuan University (Chengdu, China).

### Biological enrichment and immunologic landscape analysis

RNA sequencing (RNA-seq) expression data, along with corresponding clinical data of PDAC were collected from The Cancer Genome Atlas (TCGA) database and Gene Expression Omnibus (GEO) database. (GSE183795, GSE62452) For functional enrichment analysis, we employed the GSVA algorithm, along with GO and KEGG enrichment analyses. BEST, a web application designed to thoroughly explore biomarkers in solid tumors using large-scale data, was also used for functional enrich analysis, survival analysis and immunotherapy (Liu et al. 2023). To identified the activation of cell signaling pathways in high and low GGCT expression groups, PROGENy (Pathway RespOnsive GENes for activity inference) was used in our study (Schubert et al. 2018). We also utilized the ssGSEA algorithm to analyze immune cell infiltration and functionality. Additionally, the Tumor Immune Dysfunction and Exclusion (TIDE) algorithm was employed to evaluate immunotherapy response of PDAC patients (Jiang et al. 2018).

### Prognosis signature developed by machine learning algorithms

We collected GGCT-derived genes based on the expression profile of PDAC in TCGA database. A univariate Cox regression analysis was performed to identify the genes significantly correlated with prognosis of PDAC patients. We employed the LASSO algorithm to identify hub genes, which were subsequently utilized to construct a prognostic model.

### Statistical analysis

GraphPad Prism 9.5 and R (4.3.1) were used for all the statistical analysis. Data were expressed as mean ± SD, and Student's *t* test was employed for p-value calculation. For the analysis of clinicopathologic parameters and quantification of immunohistochemistry (IHC) signals, chi-square tests and Fisher's exact tests were utilized to identify statistically significant associations with GGCT expression. Univariate and multivariate survival analyses were conducted using Cox proportional hazards models. *p < 0.05, **p < 0.01, ***p < 0.001.

## Result

### The expression profile of GGCT varies from pan-cancer to pancreatic cancer

**Fig. 1:**
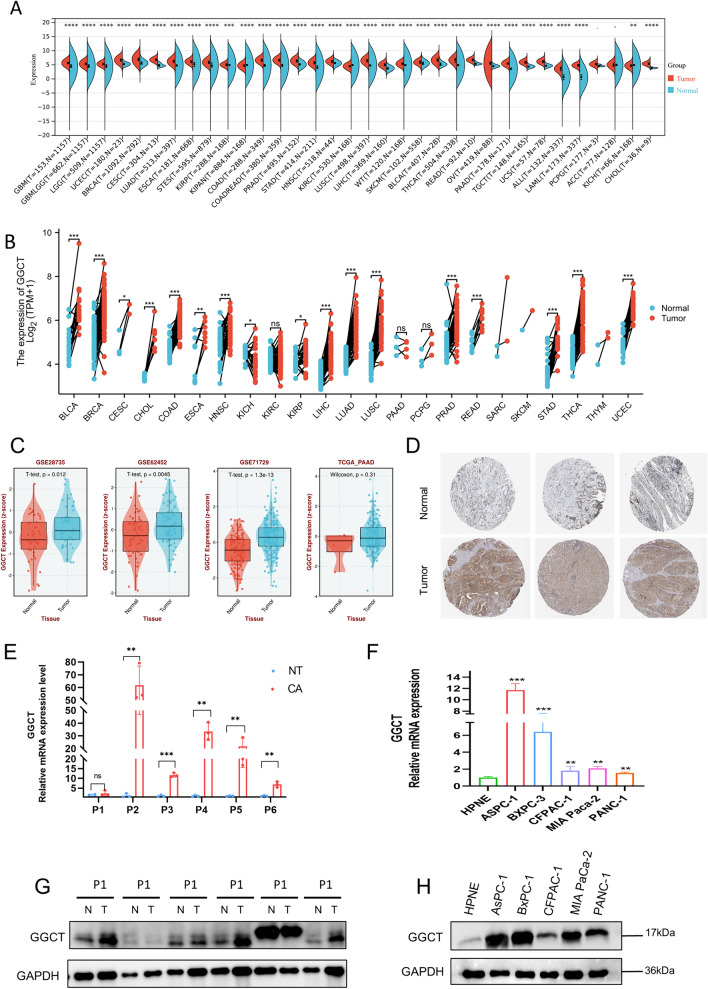
The expression profile of GGCT across pan-cancer, with a focus in pancreatic cancer. **A** Relative mRNA expression of GGCT in various cancer types. **B** Relative mRNA expression of GGCT in paired samples of various tumors. **C** GGCT exhibits high expression in tumor tissues across multiple pancreatic cancer datasets. **D** Immunohistochemical images depicting GGCT in PDAC from the HPA database. **E** Relative mRNA expression of GGCT in tumor and paired normal tissues from 6 patients with pancreatic ductal adenocarcinoma. (blue bar: normal tissue; red bar: tumor) **G** The protein expression level of GGCT in normal pancreatic cell line and PDAC cell lines. **H** GGCT protein level in tumor and paired normal tissues from 6 patients with pancreatic ductal adenocarcinoma (N: normal T:tumor). *P < 0.05, **P < 0.01, ***P < 0.001

The expression landscape of GGCT in pan-cancer is depicted in Fig. 1A, B, revealing an aberrant expression pattern in the majority of tumor, underscoring its significant role in the development of multiple tumors. The expression levels of GGCT were consistently elevated in most tumor tissue compared to the corresponding normal tissue controls, based on the TCGA and GTEx database. In paired tumor tissue and normal tissue samples across various tumors, GGCT also exhibited higher expression levels in the tumor tissue. Then we specifically investigate the expression profile of GGCT in PDAC patients, multiple datasets demonstrate that GGCT is highly expressed in pancreatic tumor tissue compared to normal pancreatic tissue (Fig. 1C). Furthermore, the immunohistochemical result we obtained from HPA database showing that GGCT exhibits higher protein expression than normal pancreatic tissue in pancreatic cancer (Zheng et al. 2021). To confirm the accuracy of the online database analysis results, we conducted verification using samples collected at West China Hospital. The mRNA expression level of GGCT is markedly elevated in tumor tissues compared to paired normal tissue (Fig. 1E). Likewise, higher protein level of GGCT was observed in paired PDAC patient samples (Fig. 1G). Additionally, in pancreatic cancer cell lines, including ASPC-1, BXPC-3, CFPAC-1, MIA Paca-2, and PANC-1, the mRNA and protein expression levels of GGCT are elevated compared to those in normal pancreatic cell lines such as hTERT-HPNE (Fig. 1F, H).

### The clinical significance of GGCT in patients with pancreatic cancer

**Fig. 2:**
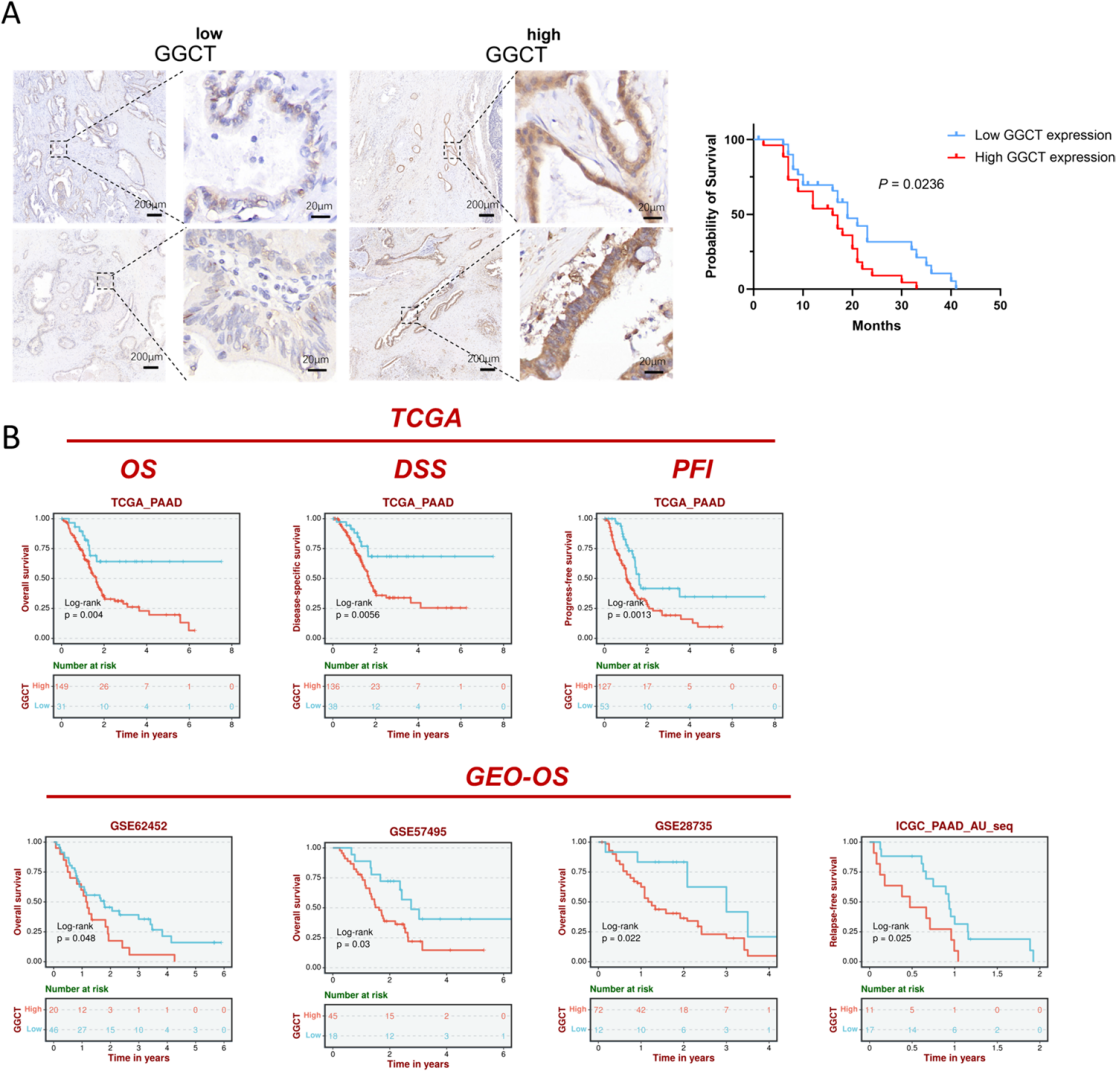
IHC and survival analysis on PDAC patients to assess the association between GGCT expression levels and survival outcomes. **A** Immunohistochemical staining images portraying low GGCT expression and high GGCT expression in pancreatic ductal adenocarcinoma tissues. **B** Prognosis performance of GGCT in multiple PDAC databases

The differential expression of GGCT in normal pancreatic tissue, pancreatic cancer tissue, and corresponding cell lines suggests that GGCT likely plays a crucial role in the occurrence and development of pancreatic cancer. Therefore, investigating the clinical significance of GGCT in patients with pancreatic cancer is essential. To evaluate the prognosis performance of GGCT in PDAC patients, we used the clinical specimens collected from West China Hospital to conducted the IHC assay. The results demonstrated significant variations of the GGCT immunostained signals in the PDAC samples. Kaplan–Meier analysis revealed a positive correlation between higher GGCT expression level and poor overall survival in patients with pancreatic cancer. (Fig. 2A) Analysis of the TCGA database indicates a correlation between elevated GGCT expression and diminished overall survival (OS), disease-free survival (DSS) and progression-free survival (PFI) in PC patients. Concurrently, various GEO and ICGC cohorts suggest that heightened GGCT expression in pancreatic cancer patients is linked to inferior OS and recurrence-free survival (RFS) (Fig. 2B).

Subsequently, we explored the expression patterns of GGCT and its correlation with clinicopathological characteristics in a separate set of pancreatic cancer samples. Table 1 indicating that a total of 57 patients were involved in the analysis, with 26 showing high expression of GGCT and 31 showing low expression. Statistical analysis revealed that the expression of GGCT is not significantly associated with Age (*p* = 0.681), Sex (*p* = 0.353), Lymph node metastasis (*p* = 0.707), tumor grade (*p* = 0.581), preoperative CA199 value (*p* = 0.652), and preoperative CEA value (*p* = 0.678). Univariate Cox proportional hazards analysis for survival of PAAD patients in According to Table 2, the expression level of GGCT (*p* = 0.032) was found to be a statistically significant risk factor affecting the clinical survival of patients diagnosed with pancreatic adenocarcinoma. To further enhance the reliability of the results, we conducted multivariate analysis including tumor grade, which is considered a crucial factor in clinical settings associated with patient survival. It turns out GGCT expression level remains as significant prognostic markers for clinical outcomes in patients (Table 3), suggesting that GGCT could potentially be used as a prognostic indicator for pancreatic cancer prognosis. At the same time, the prognostic value of GGCT in various tumors was illustrated in Supplementary Fig. 1.

**Table 1:** Association of GGCT expression and clinicopathologic parameters

Parameters		*n*	Low expression	High expression	*X*^2^	*P* value
Number of patients	57	31	26			
Age	≤ 60	28	16	12	0.169	0.681
	> 60	29	15	14		
Sex	Male	39	20	19	0.863	0.353
	Female	18	11	7		
TNM stage	I–II	55	30	25	NA	1.00
	III–IV	1	1	0		
	Loss	1				
Lymph node metastasis	No	38	20	18	0.141	0.707
	Yes	19	11	8		
Grade	Well and moderate	19	11	8	0.305	0.581
	Poor	34	17	17		
	Loss	4				
Nerve invasion	Yes	40	22	18	0.012	0.912
	No	15	8	7		
	Loss	2				
Smoke	Yes	25	10	15	2.85	0.091
	No	32	20	12		
Drink	Yes	21	5	16	12.531	< 0.001^*^
	No	36	26	10		
Hypertension	Yes	14	7	7	1.44	0.704
	No	43	24	19		
Diabetes	Yes	7	5	2	0.934	0.334
	No	50	26	24		
Preoperative CA19-9 value	< 37	18	9	9	0.204	0.652
	≥ 37	39	22	17		
Preoperative CEA value	< 5	41	23	18	0.172	0.678
	≥ 5	16	8	8		

^*^P < 0.05 was considered statistically significant

**Table 2:** Univariate Cox proportional hazards analysis for survival of PDAC patients

Variable	Hazard ratio	95% confidence interval	*P* value
GGCT expression (low/high)	0.510	0.276–0.943	0.032^*^
Age (≤ 60/ > 60)	0.713	0.398–1.280	0.257
Gender (female/male)	0.693	0.372–1.292	0.249
TNM stage (I–II/III–IV)	3.778	0.490–29.107	0.202
Grade (poor/moderate and well)	1.024	0.534–1.962	0.943
Nerve invasion (no/yes)	0.633	0.324–1.236	0.181
Lymph node metastasis (no/yes)	1.738	0.915–3.299	0.091
Preoperative CA19-9 value (< 37/ ≥ 37)	0.951	0.510–1.774	0.967
Preoperative CEA value (< 5/ ≥ 5)	0.891	0.482–1.649	0.714

^*^P < 0.05 was considered statistically significant

**Table 3:** Multivariate Cox proportional hazards analysis for survival of PDAC patients

Variables	Hazard ratio	95.0% CI	*P* value
Grade	1.385	0.681–2.817	0.369
GGCT expression	0.482	0.241–0.962	0.038^*^

^*^P < 0.05 was considered statistically significant

### The role of GGCT in tumor immune regulation revealed by analysis from pan-cancer to pancreatic caner

**Fig. 3:**
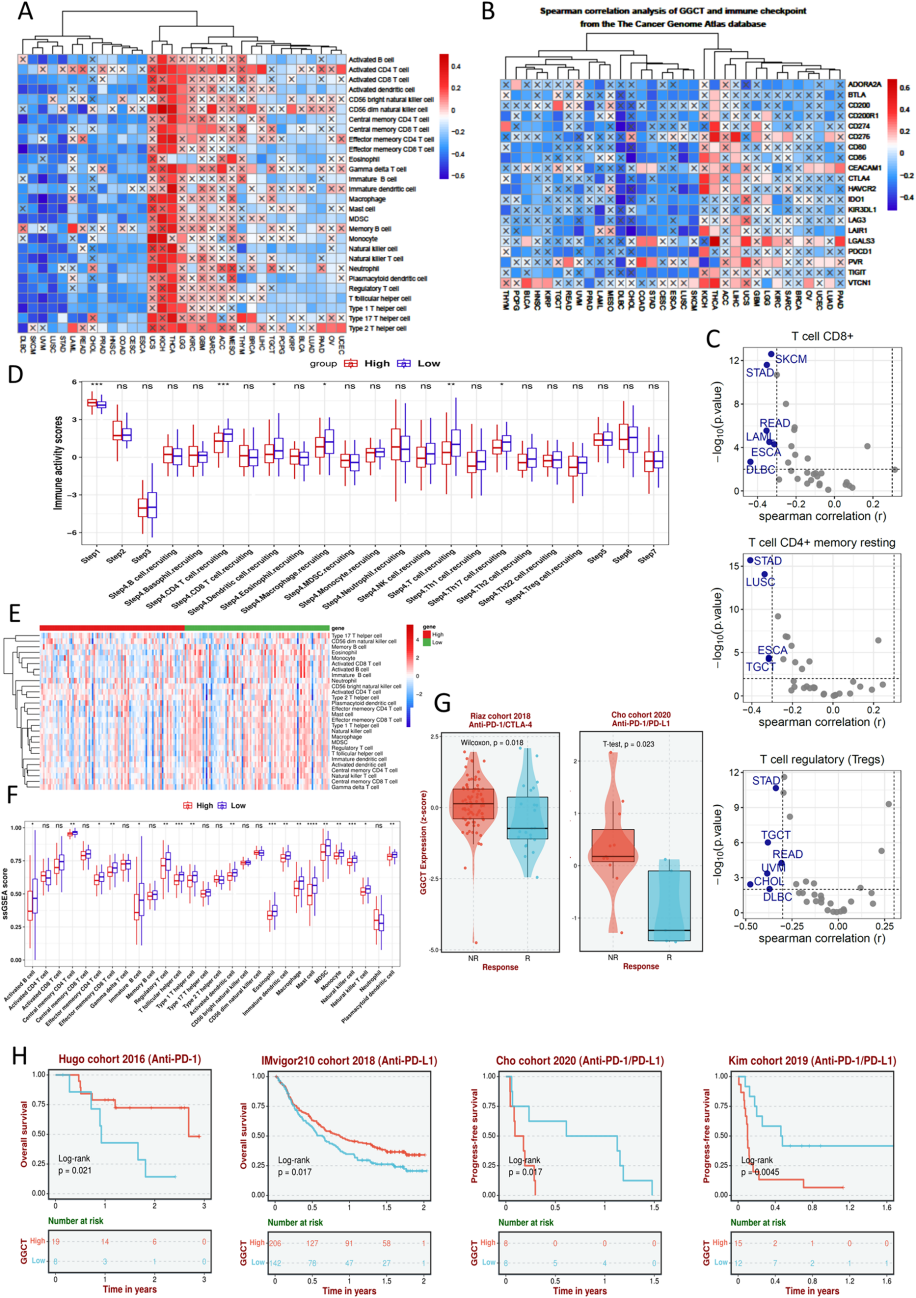
Assessing the immunological significance of GGCT in pan-cancer and pancreatic cancer by ssGSEA analysis and online database tools (best). **A** Correlation analysis between GGCT and immune cell infiltration across various cancer types. **B** Spearman correlation analysis of GGCT and immune checkpoint in pan-cancer. **C** Correlation analysis of GGCT and T cell CD8 + , T cell CD4 + memory reating and T cell regulatory (Tregs). **D** Immune activity scores of high and low GGCT expression groups in PDAC patients. **E** Heatmap of immune cell infiltration in high and low GGCT expression groups in PDAC patients. **F** Box plot of immune cell infiltration in high and low GGCT expression groups in PDAC patients by ssGSEA analysis. **G** Differences in immunotherapeutic responsiveness between the high and low GGCT expression groups. **H** Survival outcomes in the immunotherapy cohort between the high and low GGCT expression groups. *P < 0.05, **P < 0.01, ***P < 0.001

We firstly investigated the correlation between GGCT and immune cell infiltration across various cancer types by ssGSEA, and found that the association between GGCT and immune cell infiltration differs across various tumors (Fig. 3A). It demonstrates a positive correlation in tumors like THCA and UCS, whereas it presents a negative correlation in quite a few cancer types, including STAD, COAD, PAAD, ect. For instance, we found that the expression of GGCT is negatively correlated with CD8^+^ T cells, as well as CD4 + memory resting and T cell regulatory in STAD, LUSC, READ, and various other tumors ((Fig. 3C). Then we analyzed the association of GGCT with pan-caner immune checkpoint, and the results shows that GGCT is negatively related to a wide range of immune checkpoint genes in various tumors (Fig. 3B). Moreover, we assessed the immune activity scores of PDAC patients in high and low GGCT expression groups, and found that patients with low GGCT expression exhibit stronger activity in CD4 + T cell recruiting, dendritic cell recruiting, macrophage recruiting and etc. (Fig. 3D). Then we investigated the relationship between GGCT and immune-infiltrating cells in pancreatic cancer (Fig. 3E). The findings revealed an inverse correlation between elevated GGCT expression and the predominant immune cell population. Figure 3F exhibits outcomes analyzed using the ssGSEA algorithm, akin to the heatmap depicted earlier. The results of the analysiscohort imply a potential correlation between GGCT and immune suppression in cancer patients, particularly those diagnosed with pancreatic cancer. Following this, we explored the immune treatment response and prognosis in pancreatic cancer patients exhibiting both high and low levels of GGCT expression. The patients with elevated GGCT expression in both the Riaz cohort 2018 and Cho cohort 2020 were indeed confirmed to exhibit a poorer immune treatment response to Anti-PD-1/CTLA-4 (Fig. 3G). Multiple immune treatment cohorts, including the Hugo Cohort 2016, IMivigor210 Cohort, and others, consistently reveal significantly poorer overall survival (OS) in PDAC patients with high GGCT expression (Fig. 3H).

### GGCT exerts broad regulatory effects on biological processes in PDAC

**Fig. 4:**
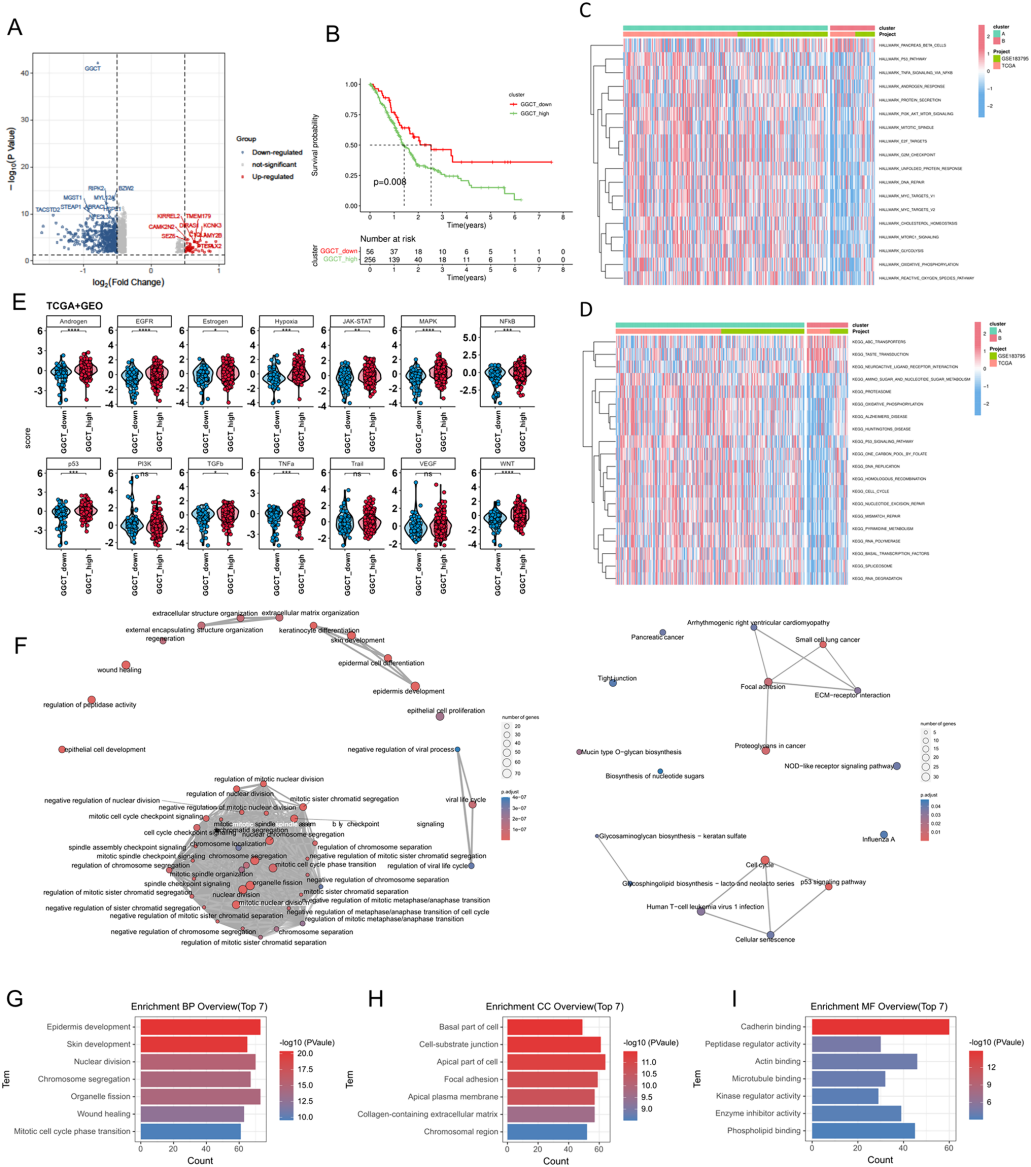
The biological function of GGCT in PDAC patients. **A** Volcano plot displaying DEGs between high and low GGCT expression. **B** Survival analysis of PDAC patients in high and low GGCT expression groups. **C**,**D** GSVA analysis of KEGG and hallmark gene set. **E** The activation of signaling pathways in high and low GGCT expression subtypes, assessed by PROGENy algorithm. **p < 0.01; ***p < 0.001. **F** Correlation between GO and KEGG enrichment analysis of DEGs. **G**–**I** GO enrichment analysis of DEGs

Based on the median expression level of GGCT, we divided 319 pancreatic cancer patient samples collected from TCGA and GEO into two groups of high and low expression, and identified the differentially expressed genes (DEGs). Figure 4A shows the significant up-regulated and down-regulated genes in both groups. The survival analysis results are consistent with the previous description, indicating a correlation between high GGCT expression and poorer prognosis (Fig. 4B). GSVA functional enrichment analysis in Fig. 4C reveals that P53-pathway, TNFA_signaling via NF-kB, PI3K_AKT_MTOR_signaling, DNA_REPAIR and others were enriched in high GGCT expression group. To explore the inherent biological activity differences between the two subtypes, we utilized PROGENy (Pathway RespOnsive GENes for activity inference) to evaluate the activation levels of cell signaling pathways in each subtype. The establishment of our study indicated that there were statistically significant distinctions in multiple signaling pathways, such as EGFR, WNT, and others, implicated in tumorigenesis between the groups with high and low expression levels (Fig. 4D). Then we visualized the correlation analysis between the enriched gene sets and pathways, and the results are shown in Fig. 4H. According to the Gene Ontology (GO) analysis, the top enriched terms for Biological Process (BP) included cell-substrate junction, focal adhesion (Fig. 4E). For Cellular Component (CC) the enriched terms included Cadherin binding, Microtubule binding, ect (Fig. 4F). Nuclear division, chromosome segregation, etc., were involved in Molecular Function (MF) (Fig. 4G).

### A prognosis signature identified through machine learning using molecules derived from GGCT

**Fig. 5:**
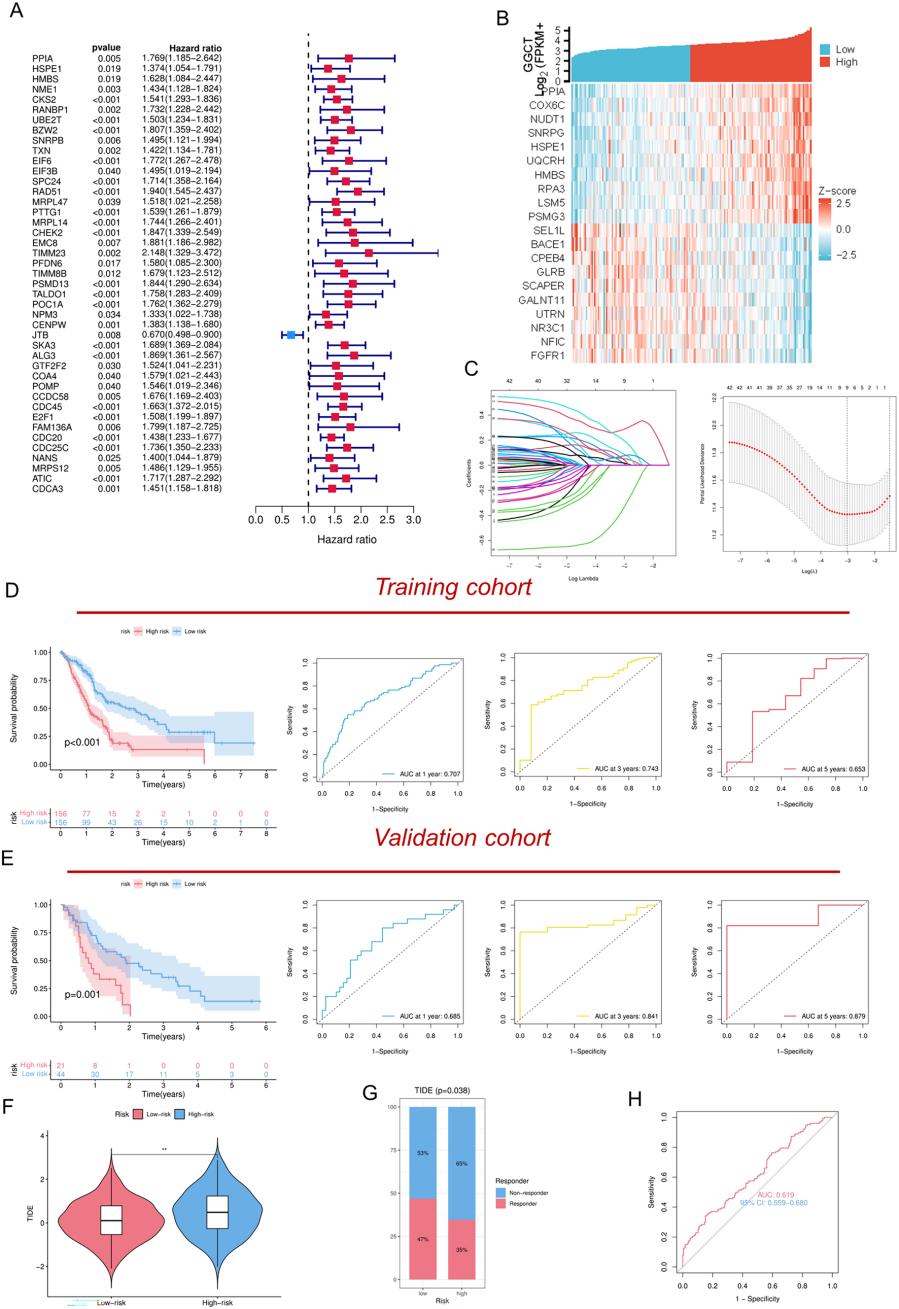
Development of a prognosis signature based on the LASSO regression analysis from MYBL1-derived genes. **A** Cox regression analysis identified to identify molecules associated with the survival of PDAC patients. **B** The heatmap illustrates the distribution of top 10 co-expressed genes with GGCT. **C** LASSO regression analysis for selecting signature genes. **D** The efficacy of our signature in predicting patient survival within the training cohort. **E** The efficacy of our signature in predicting patient survival within the validation cohort. **F**, **G** TIDE scores of high and low risk groups. **H** The performance of our risk signature in predicting immunotherapy sensitivity

Utilizing the GGCT expression levels in PDAC patients, we identified 100 genes that exhibited a positive correlation with GGCT in the TCGA database. The distribution of top 10 co-expressed genes with GGCT was illustrated in Fig. 5B. Then we conducted univariate Cox regression analysis to identify prognosis-related molecules with *P* < 0.05 (Fig. 5A). To further investigate the clinical significance of these genes, we employed the LASSO machine learning method to construct a relevant prognostic model and identify best variables (Fig. 5C). Based on the formular (Risk score = Expression _NME1_*Coef _NME1_ + Expression _BZW2_*Coef _BZW2_ + Expression _SPC24_*Coef _SPC24_ +  + Expression _RAD51_*Coef _RAD51_ +  + Expression _MRPL14_*Coef _MRPL14_ + Expression _PFDN6_*Coef _PFDN6_ + Expression _JTB_*Coef _JTB_ + Expression _ALG3_ *Coef _ALG3_ + Expression _CDC45_ *Coef _CDC45_), we calculated risk scores for each sample and divided them into high-risk and low-risk groups. Survival analysis indicated that within the training cohort, individuals classified into the high-risk group may experience worse OS. Our predictive model also demonstrated good forecasting performance, with a 1-year AUC of 0.707; 3-year AUC of 0.734; 5-year AUC of 0.653 (Fig. 5D). At the same time, our signature exhibited satisfactory performance in an external validation cohort (Fig. 5E). Considering the previous analyses, the expression of GGCT may be negatively correlated with the immunotherapy response in pancreatic cancer patients. Next, we assessed the immunotherapy response in PC patients by calculating the TIDE scores for the high-risk and low-risk groups. Similar to our hypothesis, the results indicate that patients in the high-risk group have a lower rate of immunotherapeutic response (Fig. 5F–H).

### Suppressing GGCT hinders the proliferation and migration of PDAC cells in vitro and in vivo

**Fig. 6:**
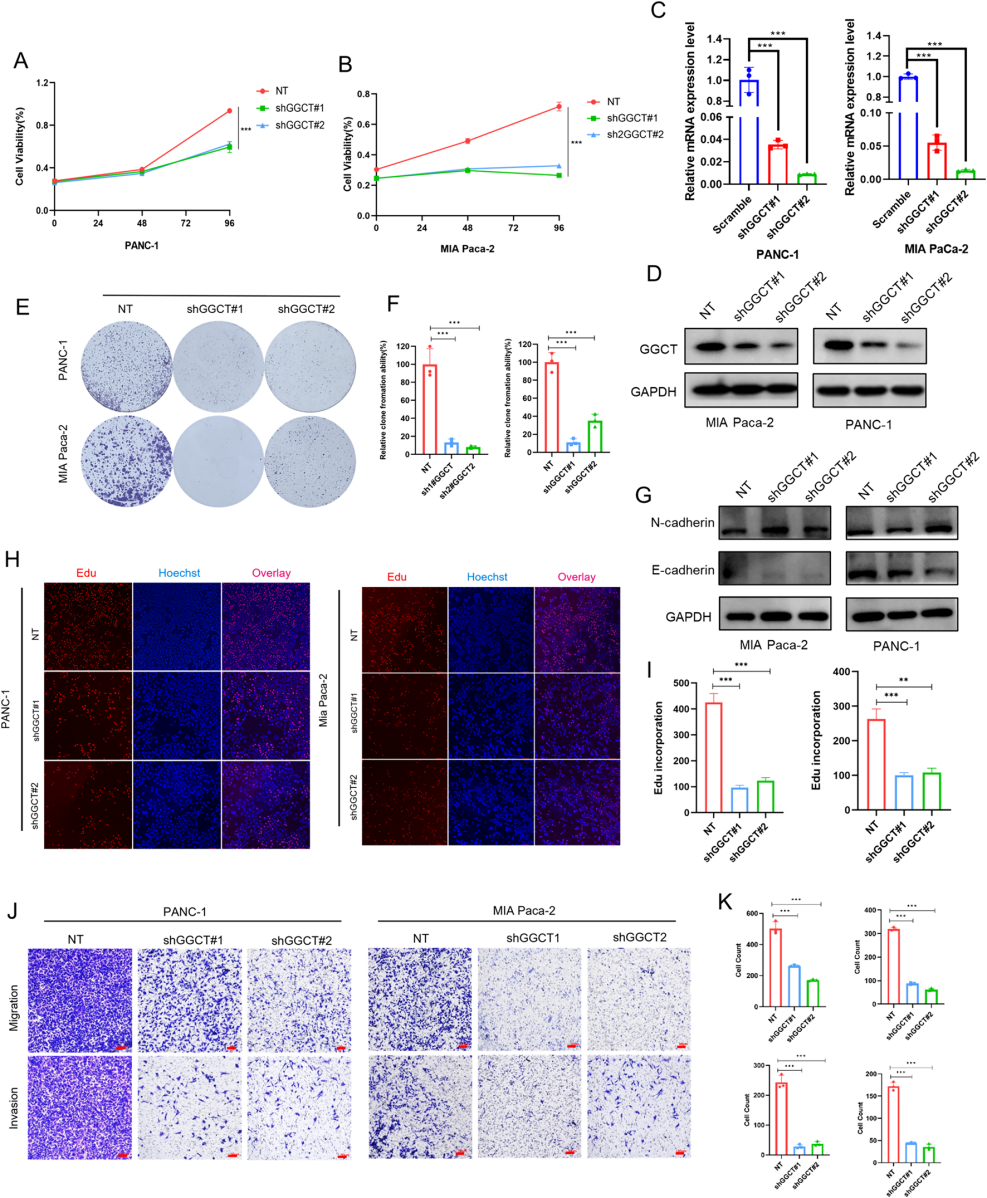
Knocking down GGCT impedes the growth and migration of PDAC in vitro. **A**, **B** Assessment of cell proliferation in PANC-1 and MIA PaCa-2 following GGCT knockdown using CCK-8 assays. **C** mRNA levels after knocking down GGCT in PANC-1 and Mia Paca-2. **D** Western blot results demonstrated the knockdown effect of GGCT in PANC-1 and MIA Paca-2 cells. **E**, **F** Colony formation Measuring cell growth of PANC-1 and MIA Paca-2 cells with GGCT knockdown. **G** Westeren blot indicating EMT protein markers after downregulate GGCT in PANC-1 and MIA Paca-2 **H**, **I** Detecting cellular proliferation activity through the Edu assay. **J**, **K** Examining the migratory capacity of PANC-1 and MIA PaCa-2 cells with GGCT knockdown by performing Migration and Invasion assays. *P < 0.05, **P < 0.01, ***P < 0.001

**Fig. 7:**
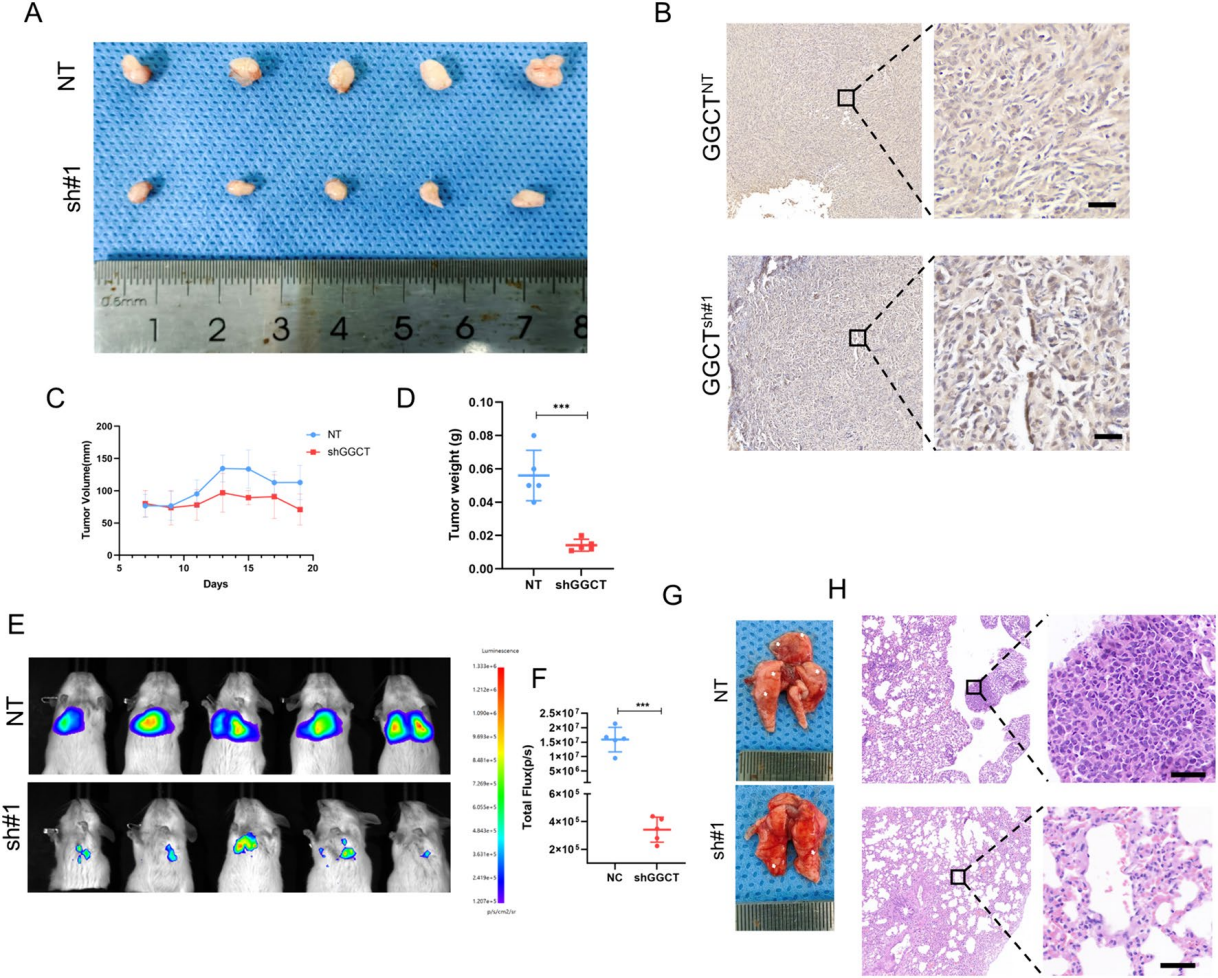
Suppressing GGCT hinders the growth and migration of PDAC in vivo. **A** Representative image displaying isolated PANC-1 tumor xenografts from mice in cohorts. **B** IHC image of GGCT in subcutaneous tumor from mice. **C** The tumor weight of isolated tumor from mice model. **D** The growth curve of subcutaneous tumors in mice monitored every two days. **E**, **F** NCG mice were injected with Luc-labeled PANC-1 cells via tail vein injection, and luciferase activity was visualized 15 days after transplantation. **G** The lung images of mice display the status of lung metastasis. **H** HE stain of the lug issure. *P < 0.05, **P < 0.01, ***P < 0.001

The CCK-8 assay revealed a significant reduction in the proliferation capacity of PDAC cells upon the notable inhibition of GGCT (Fig. 6A, B).The effective knockdown of GGCT in Panc-1 and Mia Paca-2 cells was confirmed in Fig. 6C, D. The colony formation assay confirmed the consistency of the observed pattern (Fig. 6E, F). Following that, we examined the levels of EMT marker proteins, including E-cadherin and N-cadherin (Fig. 6G). Moreover, a decreased proportion of EdU-positive cells was noted in cells with GGCT knockdown (Fig. 6H, I). Through migration and invasion assays, we found that knocking down GGCT significantly inhibited the progression of pancreatic cancer cells (Fig. 6J, K). Then, we investigated the impact of GGCT on the progression of pancreatic cancer cells in vivo using xenograft models and mouse model of lung metastasis. After inoculating PANC-1 cells subcutaneously into mice, we observed that tumors in the GGCT knockdown group were significantly smaller than those in the NC group (Fig. 7A–D).

Figure 7B showed the IHC image of GGCT in subcutaneous tumor from mice. We also established a mouse lung metastasis model to assess the impact of GGCT on pancreatic cancer metastasis capability. In vivo bioluminescence imaging revealed a notable decrease in both lung fluorescence intensity and the count of metastatic lungs within the GGCT knockdown group compared to the control group (Fig. 7E–H). Additionally, the lung imaging and total fluorescence was shown in Supplementary Fig. 2. These findings imply that GGCT significantly contributes to the advancement of pancreatic cancer both in vitro and vivo.

### GGCT hinders the advancement of pancreatic cancer by inducing the upregulation of c-Myc

**Fig. 8:**
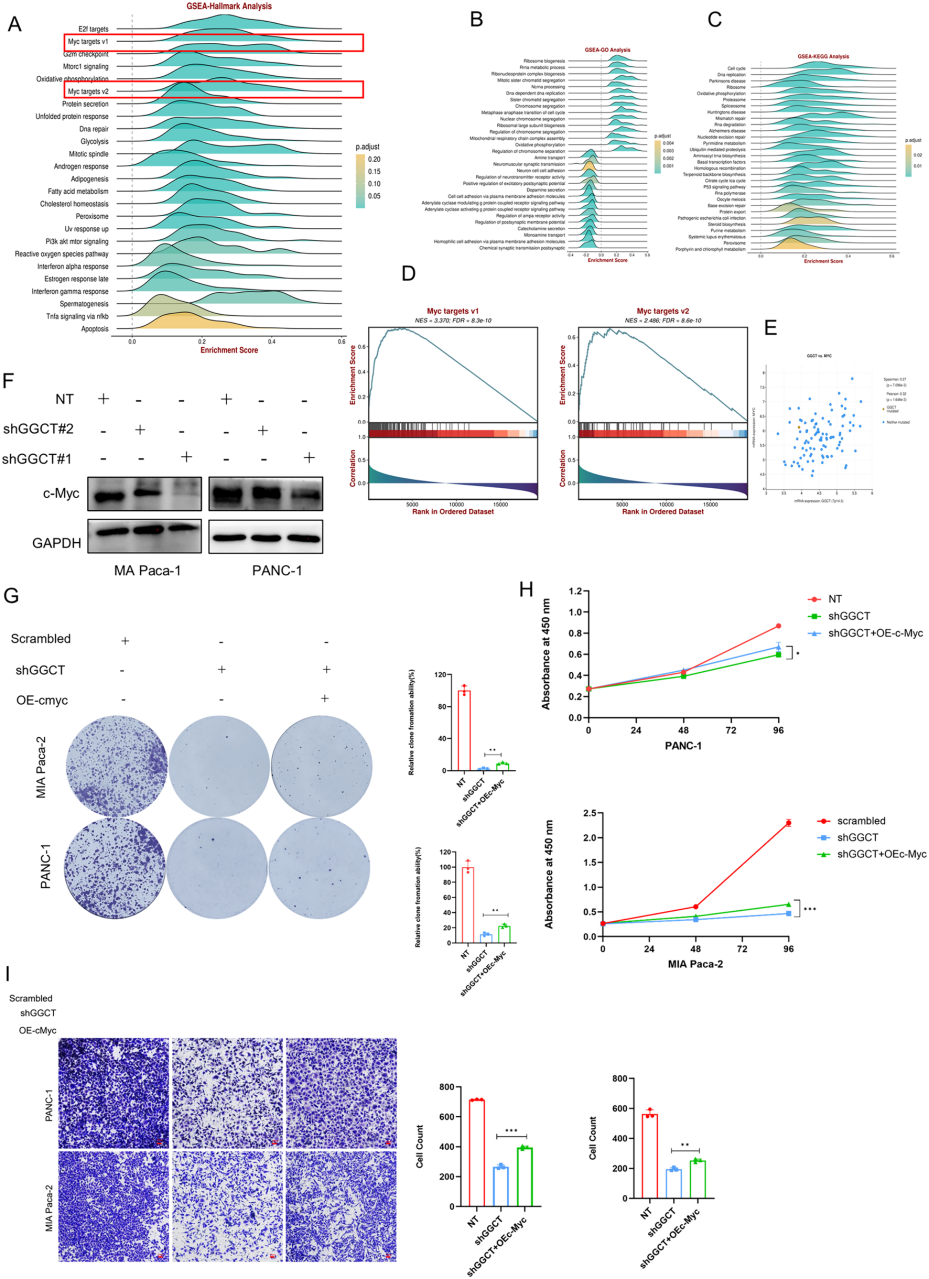
GGCT hinders the advancement of pancreatic cancer by inducing the upregulation of c-Myc. **A** GSEA enrichment analysis of hallmark gene sets for GGCT in multiple pancreatic cancer datasets. **B** GSEA analysis of GO enrichment for GGCT. **C** GSEA analysis of KEGG enrichmen for GGCT. **D** GSEA analysis of c-Myc releted gene set for GGCT. **E** Correlation analysis of GGCT and c-Myc in PDAC. **F** Western blot of c-Myc in PANC-1 and MIA PaCa-2 cells with or without GGCT knockdown. **G** Cell proliferation determined by Colony assays in GGCT knockdown cells with or without overspression c-Myc. **H** Cell proliferation determined by CCK8 assays in GGCT knockdown cells with or without overspression c-Myc. **I** Assessing the progression capability of cells through Migration assays in GGCT knockdown cells with or without overspression c-Myc. *P < 0.05, **P < 0.01, ***P < 0.001

Our study has already indicated that the malignant biological behaviors of PDAC are augmented by GGCT, especially attenuating the progression of PC cells. To further discuss the mechanism behind, we used online tool to conducted a GSEA analysis of Hallmark gene set, results showed that Myc targets v1 and Myc targets v2 were enriched (Fig. 8A). The GSEA-GO analysis primarily enriched in terms related to DNA dependent DNA replication, Oxidative phosphorylation, mitotic sister chromatid segregation and others. repair and other pathways (Fig. 8B). The GSEA-KEGG analysis predominantly showed enrichment in categories associated with Cell cycle, RNA degradation, P53pathway, and various other pathways (Fig. 8C). Figure 8D showing the GSEA enrichment results of Myc targets v1 and Myc targets v2. Furthermore, the results of correlation analysis indicate a positive correlation between GGCT and the *MYC* gene (*R* = 0.27/0.32, *p* < 0.001) (Fig. 7E). Subsequently, we validated the aforementioned findings in pancreatic cancer cells by knocking down GGCT. Western blot results revealed a corresponding decrease in c-Myc expression upon GGCT knockdown (Fig. 8F). To examine whether GGCT plays a role in the progression of pancreatic cancer via c-Myc, we conducted rescue experiments. The CCK8 results demonstrated that the overexpression of c-Myc could partly reverse the reduced cell proliferation caused by the inhibition of GGCT in PANC-1 and Mia Paca-2 cells (Fig. 8H). The clone formation experiments simultaneously confirmed the above results (Fig. 8G). Considering the role of GGCT in pancreatic cancer progression, we conducted migration rescue assays. The suppressed migration ability of pancreatic cancer cells due to knocking down GGCT was restored after overexpression of c-Myc (Fig. 8I).

## Discussion

Pancreatic cancer typically leads to dismal survival rates due to limited treatment options, primarily because most patients are diagnosed at an advanced stage of the disease (Halbrook et al. 2023). Currently, the primary therapeutic approach for pancreatic cancer patients remains surgical resection. However, a significant proportion of patients are diagnosed at an advanced stage precluding surgical intervention. Patients who experience disease progression after initial chemotherapy still face a dearth of effective treatment modalities (Del Chiaro et al. 2023). Hence, the identification of novel biomarkers for PDAC diagnosis and therapeutic alternatives holds immense significance.

The protein encoded by GGCT catalyzes the conversion of gamma-glutamyl dipeptides into 5-oxoproline, which is the second-to-last step in glutathione breakdown, potentially crucial for maintaining glutathione balance (Liu et al. 2014). Reports indicate significant accumulation of GGCT in various cancers, such as breast, gastric cancer, ovarian cancer and other cancers.(Gromov et al. 2010; Zhang et al. 2016; Li et al. 2018; Taniguchi et al. 2018) Numerous studies have indicated high expression of GGCT in tumor tissues, yet the underlying biological mechanisms of GGCT overexpression in tumor tissues remain unclear. Kageyama et al. (2018) observed a distinct difference in the structure of the GGCT promoter between normal cells (ARPE-19, IMR-90) and cancer cells (HeLa, MCF7), and noting a more relaxed chromatin structure in cancer cells compared to normal cells. He et al. (2019) reported an upregulation of GGCT expression in LUAD associated with chromosome 7p or 7p14.3 amplification, suggesting the involvement of GGCT in the oncogenic transformation during LUAD initiation. Furthermore, the metabolic reprogramming of GSH involving GGCT in tumors could also be a contributing factor to the elevated expression of GGCT across various cancers. In high grade ovarian cancer, high expression of GGCT has been confirmed to correlate with poorer OS, and GGCT facilitates the progression of ovarian cancer by activating the PI3K/AKT/mTOR pathway (Li et al. 2018). Li et al. (2022) reported that miR-205-5p direct binds to the 3′-UTR of GGCT, resulting in the transcriptional repression of GGCT expression, consequently antagonizing the pro-oncogenic impact in thyroid cancer. Shen et al. (2016) found in their study that GGCT exhibits up-regulation in both human glioma tissues and cell lines and fosters the proliferation of glioma by activating Notch-Akt signaling. All these studies suggest that GGCT is closely linked to the occurrence and progression of various types of tumors, thus the biological significance of GGCT in the advancement of pancreatic cancer warrants exploration. In our study, we initially analyzed the expression levels of GGCT in pan-cancer through online databases, and the results were consistent with previous literature, indicating high expression of GGCT across various types of tumors. Then we investigated the expression profile in PDAC, compared to normal tissue, multiple datasets demonstrate high expression of GGCT in tumor tissues. Immunohistochemical images obtained from the HPA database also reveal that the protein levels of GGCT are higher in pancreatic cancer compared to normal tissue. Clinical samples obtained from West China Hospital validate the same results.

Following this, we delved into the clinical features of GGCT in pancreatic cancer. Univariate Cox analysis revealed GGCT as an independent risk factor for pancreatic cancer patients, while survival analysis showed a connection between high GGCT expression and poorer OS in these patients. Bioinformatics analysis also indicated that GGCT is correlated with DSS (Disease Specific Survival) and PFI (Progression-Free Interval) of PC patients. Functional enrich analysis indicating that high GGCT expression group exhibit activation of multiple tumor-related signaling pathways, including EGFR, PI3K, P53 and so on, suggesting GGCT may induce PDAC progression though activation of those cell signal pathways. In vitro assays demonstrated that GGCT is up-regulated in PC cells, Suppressing GGCT markedly inhibited the proliferation and progression of pancreatic cancer cells. Furthermore, we confirmed that downregulation of GGCT led to noticeably reduced subcutaneous tumor growth in mice compared to the control group. Moreover, in a mouse model of lung metastasis, knockdown of GGCT significantly inhibited the lung metastatic potential of pancreatic cancer. In the investigation of mechanisms, we observed a decrease in c-Myc expression following the downregulation of GGCT, indicating that GGCT's suppressive effect on pancreatic cancer may be mediated through the modulation of c-Myc. Subsequently, cells with GGCT knockdown were transfected with plasmids overexpressing c-Myc, resulting in partial restoration of proliferation and invasion capabilities. c-Myc has been reported to be an oncogene in various tumors, participating in the development and progression of malignant tumors by regulating metabolism, differentiation, apoptosis and so on (Llombart and Mansour 2022). It is well-established in the research field that high expression of c-Myc promotes the progression of pancreatic cancer, many studies targeting c-Myc to suppress pancreatic cancer proliferation have been conducted (Ala 2022). We investigated the mRNA and protein levels of c-Myc after GGCT knockdown and found that both transcriptional and protein levels were decreased, suggesting that GGCT may regulate c-Myc expression through transcriptional regulation. Studies have reported that the use of PI3K inhibitors can induce a decrease in c-Myc expression in pancreatic cancer cells (Sharma et al. 2015), while the use of c-Myc inhibitors can attenuate PI3K/AKT/mTOR-induced pancreatic cancer cell proliferation. This suggests that the PI3K/AKT/mTOR pathway is an upstream signaling pathway regulating c-Myc expression in pancreatic cancer cells (Asano et al. 2004). In our study, we found enrichment of the PI3K/AKT/mTOR pathway in the differentially expressed gene functions between the high and low expression groups of GGCT in pancreatic cancer samples. Therefore, we speculate that GGCT may also regulate c-Myc expression by targeting this pathway, but further experimental validation is required. Moreover, research has shown that GGCT can modulate the expression of the transcription factor HIF-1α by regulating cellular metabolism (Taniguchi et al. 2022). We hypothesize that similar regulatory mechanisms may be present in PDAC.

The improvement of treatment strategies for pancreatic cancer remains extremely challenging (Del Chiaro et al. 2023). Despite chemotherapy being administered to patients with advanced and metastatic disease, the benefits have been limited and short-lived, providing only modest improvements (O'Reilly et al. 2019). Recent years, immunotherapy has revolutionized the landscape of cancer treatment in various solid tumors, its application in pancreatic cancer patients has gradually become a hot and challenging research focus (Bockorny et al. 2022). The advancements of immunotherapy have been closely tied to the utilization of immune checkpoint inhibitors (ICIs) (Darvin et al. 2018). The combination of chemotherapy and ICIs has demonstrated effectiveness in various types of solid cancers (Emens and Middleton 2015). Numerous such combinations have been investigated in PDAC as well (Principe et al. 2021). Xu et al. (2020) reported in their study that silencing GGCT not only affects the proliferation, invasion and migration abilities of endometrial carcinoma but also regulates the expression of PD-L1, thereby enhancing the killing capability of CD8 + T lymphocytes against tumor cells. Accordingly, we explored the regulatory role of GGCT in immunotherapy from pan-cancer to pancreatic cancer. Our study suggested that GGCT may serve as a potential immunosuppressive regulator. Firstly, our observations indicate a widespread negative correlation between GGCT and immune checkpoints in various types of tumors. Following this, we detected functional disparities in various immune cells between the high and low expression groups of GGCT in pancreatic cancer. Notably, patients with high GGCT expression demonstrated significantly diminished immune cell infiltration and functional activity, including decreased CD4 T cell recruitment, Dendritic cell recruiting, activated monocytes, macrophages, natural killer cells and so on. In recent years, targeted immunotherapy using NK cells has shown remarkable promise in the treatment of hematologic malignancies. Researchers have accelerated the exploration of NK cell application in the treatment of solid tumors, achieving some encouraging progress (Liu et al. 2021). Fitzgerald et al. (2021) Based on the above results, we speculate that GGCT may reshape the immune microenvironment of PDAC by reducing the infiltration of anti-tumor immune cytotoxic cells like NK cells, leading to poorer immune therapy response. Fitzgerald et al. (2021) found that a small molecule DPP inhibitor significantly inhibits the proliferation of PDAC tumors in mT3-2D and Pan02 subcutaneous syngeneic murine models in C57BL/6 mice, and this inhibitory effect depends on enhancing the immune infiltration of NK and CD8 + T cells, while also enhancing the efficacy of anti-PD1. Guo et al. (2022) also reported that Polysaccharide additionally observed that polysaccharides have the capacity to boost NK cell activity and suppress the proliferation of pancreatic tumors via the TLR4/MAPKs/NF-κB signaling pathway. These research findings suggest the significant role of NK cells in inhibiting the proliferation of pancreatic cancer. In our study, we observed a decrease in NK cell infiltration in patients with high expression of GGCT, suggesting a potential mechanism by which GGCT is involved in the formation of an immunosuppressive microenvironment. However, further experimental validation is still required. Of greater significance, we noted that in several pancreatic cancer immunotherapy cohorts, patients with high GGCT expression demonstrated poorer response to Anti-PD-L1/CTLA-4 treatment and worse overall survival. In conclusion, GGCT is likely involved in the remodeling of the immune microenvironment in PDAC. The variation in GGCT expression could serve as a critical factor for predicting the response to immune therapy in PDAC patients, and targeting GGCT might offer benefits for immunotherapy. On top of that, we established a prognosis signature based on molecules derived from GGCT, which exhibits strong predictive ability for patient prognosis. Additionally, it suggests that PDAC patients in high GGCT expression group exhibit inferior responses to immunotherapy and worse OS. All these findings highlight the significance of GGCT for PDAC patients.

Although we identified the expression pattern and biological functions of GGCT in PDAC via integrated analysis and in vitro and in vivo experiments, it is important to acknowledge certain limitations. Initially, it is essential to incorporate additional expression data from PDAC to validate the reliability and accuracy of our risk model. Further exploration of the inhibitory role of GGCT in PDAC progression is warranted, including an in-depth investigation into the mechanisms involving GGCT and c-Myc. Additional experimental validation is required to clarify the involvement of GGCT in immune regulation within PDAC.

### Supplementary Information

Below is the link to the electronic supplementary material.

Supplementary file1 (TIF 1279 KB)

Supplementary file2 (TIF 740 KB)

## Data Availability

The original contributions featured in this study are available within the article/Supplementary Material. For additional inquiries, please contact the corresponding author.
