# Supplementary figures and images for: Identified γ-glutamyl cyclotransferase (GGCT) as a novel regulator in the progression and immunotherapy of pancreatic ductal adenocarcinoma through multi-omics analysis and experiments
Source: J Cancer Res Clin Oncol. 2024 Jun 25;150(6):318. doi: 10.1007/s00432-024-05789-0 (PMC11196309; doi:10.1007/s00432-024-05789-0)

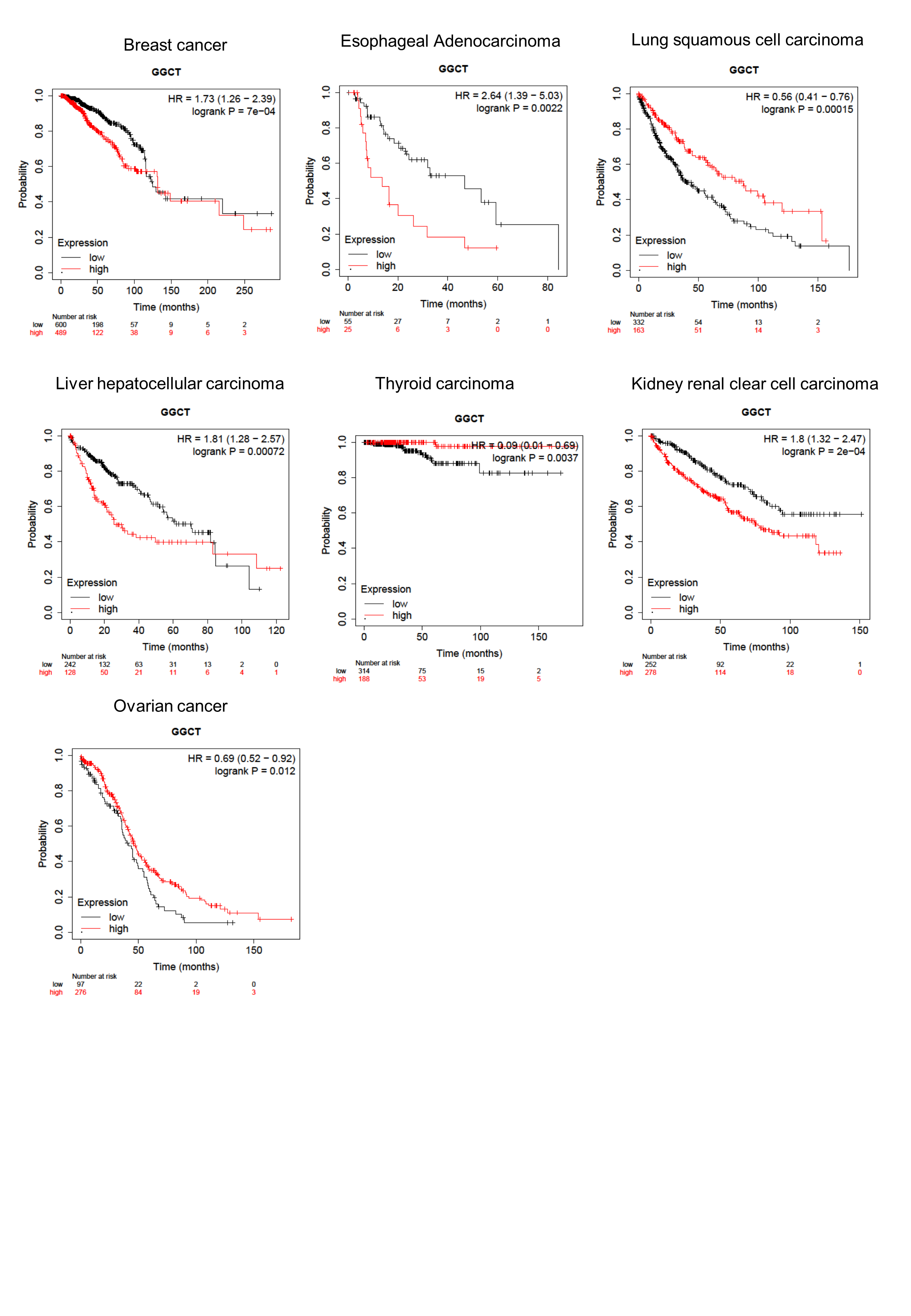

Supplement: Supplementary file 1 — Supplementary file1 (TIF 1279 KB) [file 432_2024_5789_MOESM1_ESM.tif]

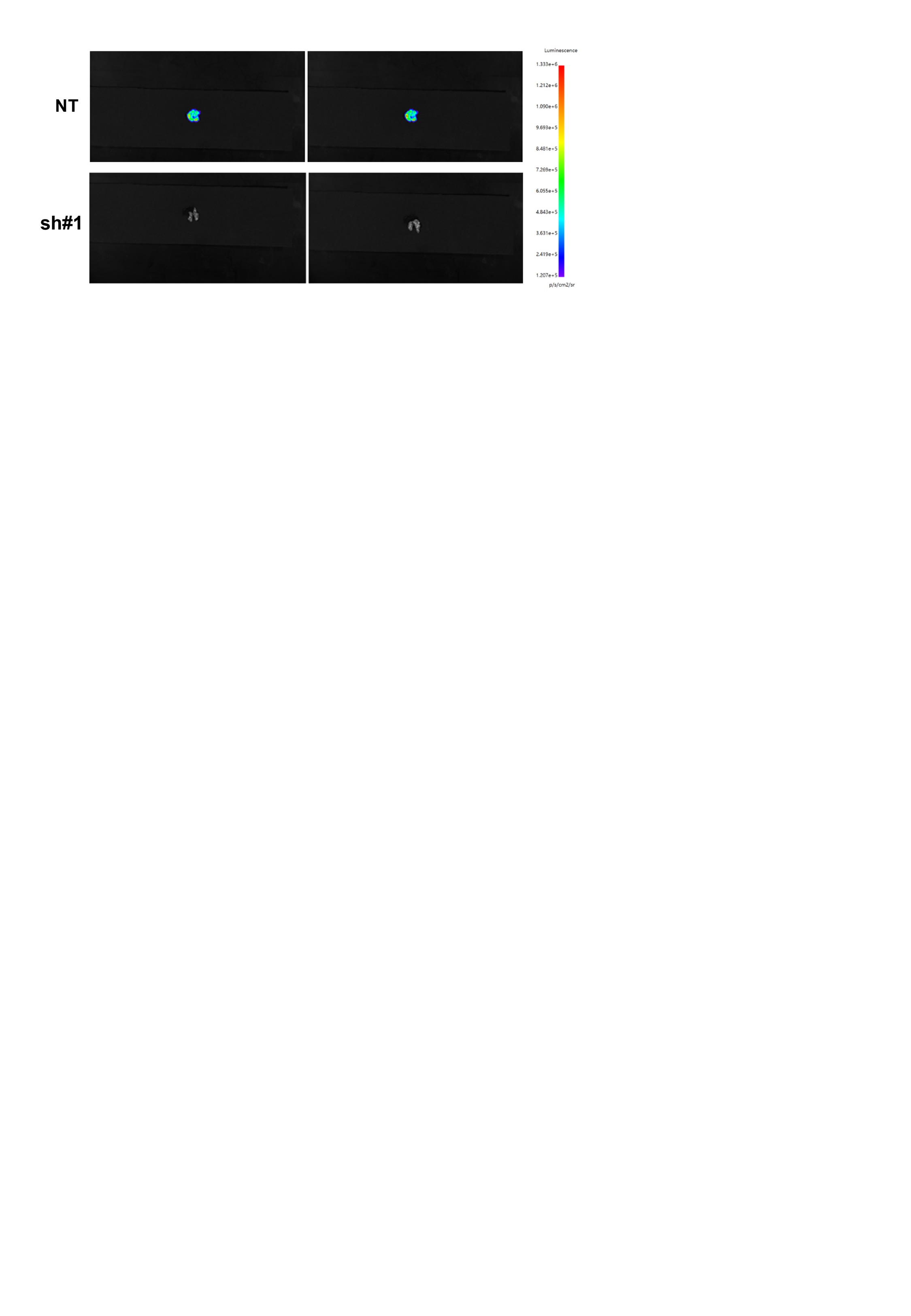

Supplement: Supplementary file 2 — Supplementary file2 (TIF 740 KB) [file 432_2024_5789_MOESM2_ESM.tif]
